# Agricultural implications of the Fukushima nuclear accident

**DOI:** 10.1093/jrr/rrw042

**Published:** 2016-08-16

**Authors:** Tomoko M. Nakanishi

**Affiliations:** Graduate School of Agricultural and Life Sciences, The University of Tokyo, Tokyo, Japan

**Keywords:** Fukushima nuclear accident, agricultural implications, ^137^Cs, soil, crop, forest, tree, animal, decontamination

## Abstract

More than 4 years has passed since the accident at the Fukushima Nuclear Power Plant. Immediately after the accident, 40 to 50 academic staff of the Graduate School of Agricultural and Life Sciences at the University of Tokyo created an independent team to monitor the behavior of the radioactive materials in the field and their effects on agricultural farm lands, forests, rivers, animals, etc. When the radioactive nuclides from the nuclear power plant fell, they were instantly adsorbed at the site where they first touched; consequently, the fallout was found as scattered spots on the surface of anything that was exposed to the air at the time of the accident. The adsorption has become stronger over time, so the radioactive nuclides are now difficult to remove. The findings of our study regarding the wide range of effects on agricultural fields are summarized in this report.

## INTRODUCTION

After the Fukushima nuclear accident, the agricultural industries, the Fukushima prefecture and the government agricultural agency commenced strategies to assist in decontamination of the agricultural land and its crops, e.g. use of potassium fertilizer or removal of the soil. The Fukushima prefecture started to measure the radioactivity of all kinds of agricultural products. Especially in the case of the rice, they inspected entire crops prior to shipment. Through these countermeasures, all of the products that were introduced into the market were below the threshold levels for safe radiation exposure.

**Fig. 1.**
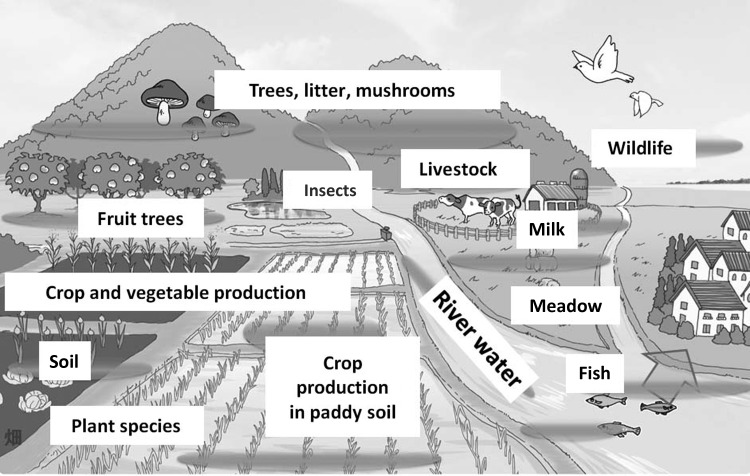
Fukushima projects conducted at our faculty.

Immediately after the accident, the Graduate School of Agricultural and Life Sciences at the University of Tokyo created an independent team consisting of a wide range of specialists—for soil, crops, wild and domestic animals, fish, forest, etc. (Fig. 1). The team member entered the contaminated sites immediately after the accident and commenced research to monitor the radioactive materials integrated into the agricultural environment. The results of these studies have been useful in the recovery of the affected area. This report summarizes the findings of our group.

## ONTAMINATION

**Fig. 2.**
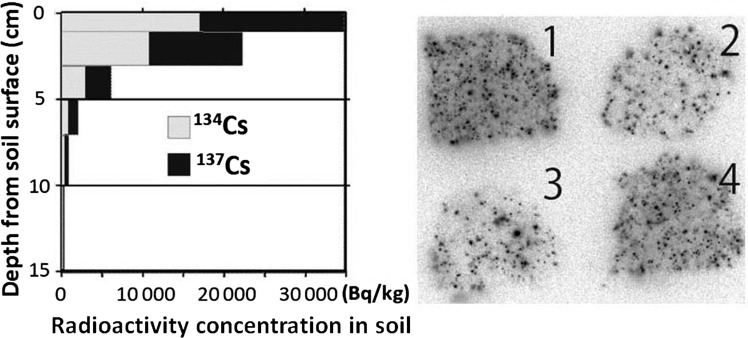
Radioactive Cs in soil [1,2]. Radioactivity measurement and soil collection was performed at the Fukushima Agricultural Technology Center at Koriyama 21 April 2011, located ∼50 km from the site of the accident. Left: The ^134^Cs and ^137^Cs radioactivity profile of the soil: soil contamination was limited to the top few centimeters of the field. Right: Autoradiography of the soils: ∼15 g of soil was collected from each of four kinds of field at the Center and spread on paper, and autoradiography was performed on an imaging plate with a 24-h exposure. (1) Farming land, 50 000 Bq/kg; (2) soil for vegetables, 8000 Bq/kg; (3) soil for wheat, 7000 Bq/kg; (4) paddy soil, 37 000 Bq/kg (Sho Shiozawa *et al*.)

The fallout was found as scattered spots on the surface of everything that was exposed to the air at the time of the accident (Fig. 2) [1,2]. The salient feature, with regard to the fallout, is that the radioactive Cs has largely remained at the initial contact site, with little movement, and is difficult to remove.

The downward movement of the radioactive fallout in the soil is currently ∼1–2 mm/year, whereas in the first 3 months after the accident it moved ∼20 mm, and during the following 3 months it moved ∼6 mm. The speed of the movement is now much slower than directly after the accident.

Since the nuclear power plant accident occurred late in winter, there were no leaves on the deciduous trees, but there were leaves on the evergreen trees in the mountains, and these needle-like leaves were highly contaminated by the fallout. After a few years, these leaves fell to the ground and were gradually decomposed by microorganisms. During the decomposition process, the radioactive Cs adsorbed on the leaves was transferred to the soil and then firmly adsorbed onto the soil particles. The radioactive Cs was only adsorbed onto the very fine particles of the clay. The mineral in the clay was recently identified as weathered biotite [3].

The river water flowing from the mountain shows very low radioactivity (<10 Bq/l). It was found that the water itself was hardly radioactive at all after filtering out the clay. The amount of the radioactive Cs flowing from the mountain was in the order of 0.1% of the total fallout amount per year.

However, the radioactivity of mushrooms growing in the forest did not decrease with time to any great extent. Some of the mushrooms harvested more than 300 km from the site of the accident were found to contain ^137^Cs only, indicating that they are still collecting the global fallout of the nuclear bomb tested during the 1960s. Since the half-life of ^137^Cs, 30 years, is much longer than that of ^134^Cs, 2 years, all of the ^134^Cs from the global fallout in the 1960s was decayed out after 50 years. This means when only ^137^Cs was detected in the mushroom, the ^137^Cs found was not from the Fukushima nuclear accident. In the case of the fallout from the Fukushima nuclear accident, the initial radioactivity ratio of ^137^Cs to ^134^Cs was the same. Therefore, currently, both nuclides should be detectable in mushrooms collecting fallout from Fukushima.

**Fig. 3.**
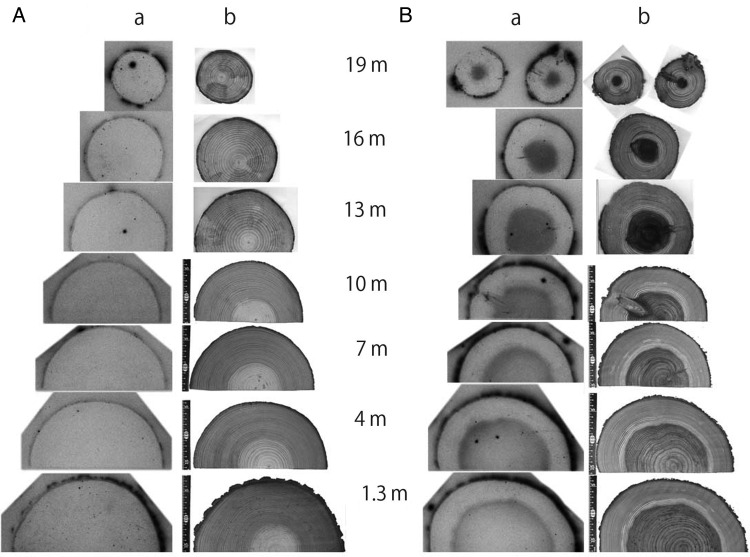
Autoradiography of trees in the forest [1,2]: (**A**) pine tree (*Pinus dinsiflora*) and (**B**) sugi (*Cryptomeria japonica*). The height of the sugi and pine tree were 23.2 m and 22.2 m, respectively. Wood disks were harvested from 1.3 m, 4.0 m, 7.0 m, 10 m, 13 m, 16 m and 19 m above the surface of the soil, and an autoradiograph of each disk was taken by an imaging plate: (**a**) autoradiograph and (**b**) photograph. The radioactivity of the wood disks was high at the surface of the bark in both trees. In the case of sugi (B), a large amount of radioactive Cs was transferred into the heartwood, especially higher in the tree. However, in the case of the pine tree (A), most of the radioactive cesium was found to be at the surface of the trunk (S. Masumori, T. Tange *et al*.).

In the case of trees, the radioactive Cs was moved directly from the surface of the trunk to the interior. Two kinds of trees in the forest were cut down in Minami–Soma City in 2012; the radioactivity of each whole tree was measured, and radiography of wood disks taken from different heights was performed (Fig. 3). Though the amount of radioactivity moved into the heartwood differed between the two kinds of tree, the contamination inside the tree was not due to radioactive Cs transport from the roots. Since the radioactive Cs was only at the surface of the soil, it was not possible for the active roots to absorb Cs. The active part of the roots for most of the trees was at least 20–30 cm below the surface of the soil, where there was no radioactive Cs.

Since rice is a staple part of the Japanese diet, the Cs-accumulating part of the grain was investigated. When the grain was milled, the radioactivity concentration was reduced to about half. Next, by washing the milled grain, the radioactivity concentration was further reduced to about half. To eat rice, water is added and steamed, which process further reduces the radioactivity to a concentration of about half that again.

During the developmental stage of the rice, from about 10 days after flowering, the radioactive Cs accumulated in the epidermis and the cereal germ of the grain. When micro-autoradiography of the germ was taken, radioactive Cs was found to be accumulated around the plumule and the radicle, suggesting that Cs was not incorporated into the newly developing tissue itself, but accumulated in the tissue surrounding the meristems, similar to the phenomenon by which the meristem is generally protected and free from heavy metals and viruses.

## RADIOACTIVE CESIUM MOVEMENT IN PLANTS

**Fig. 4.**
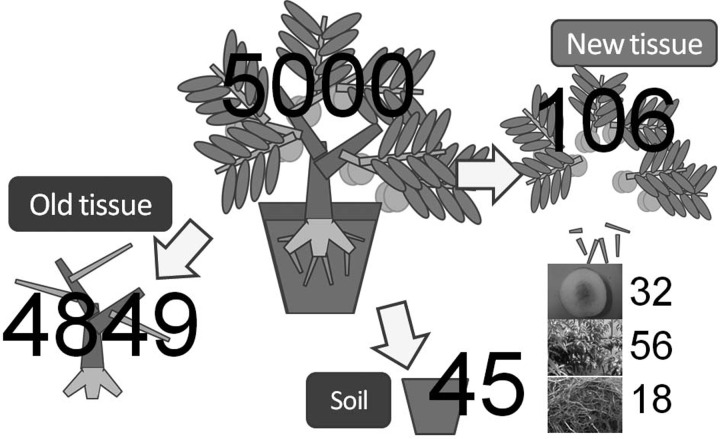
Radioactive Cs movement in a peach tree [1,2]: 5 months after the accident, about half of the radioactive Cs adsorbed onto the peach tree had been moved from the surface to inside the bark. In order to find out how the radioactive Cs incorporated in the bark moves into the newly developing tissue in the following year, a 5-year-old peach tree grown in Fukushima was chosen. After removal of the twigs, leaves and fine roots, the tree was washed well and transplanted into non-contaminated soil in Tokyo and grown for a year, developing new tissues, including fruits. When the original amount of radioactive Cs incorporated into the bark was set at 5000 Bq/kg, 4849 (97%) was not moved, but remained in the old tissue, 45 was transferred to the soil by the root activity, and 106 was transferred into the new tissue. Of the 106 Bq/kg transferred into the new tissue, the relative distributions were 32 to fruit, 56 to leaves and 18 to the roots (D. Takada *et al*.).

In order to know how radioactive Cs is transferred to the inner part of the tree in the first year, a contaminated peach tree was transplanted into non-contaminated soil after removing the twigs, leaves and fine roots. In the following year, all of the newly developed tissue (including fruits) was harvested and the radioactivity was measured [1,2]. As is shown in Fig. 4, less than 3% of the radioactive Cs accumulated inside the tree was moved to the newly developed tissue in the first year.

**Fig. 5.**
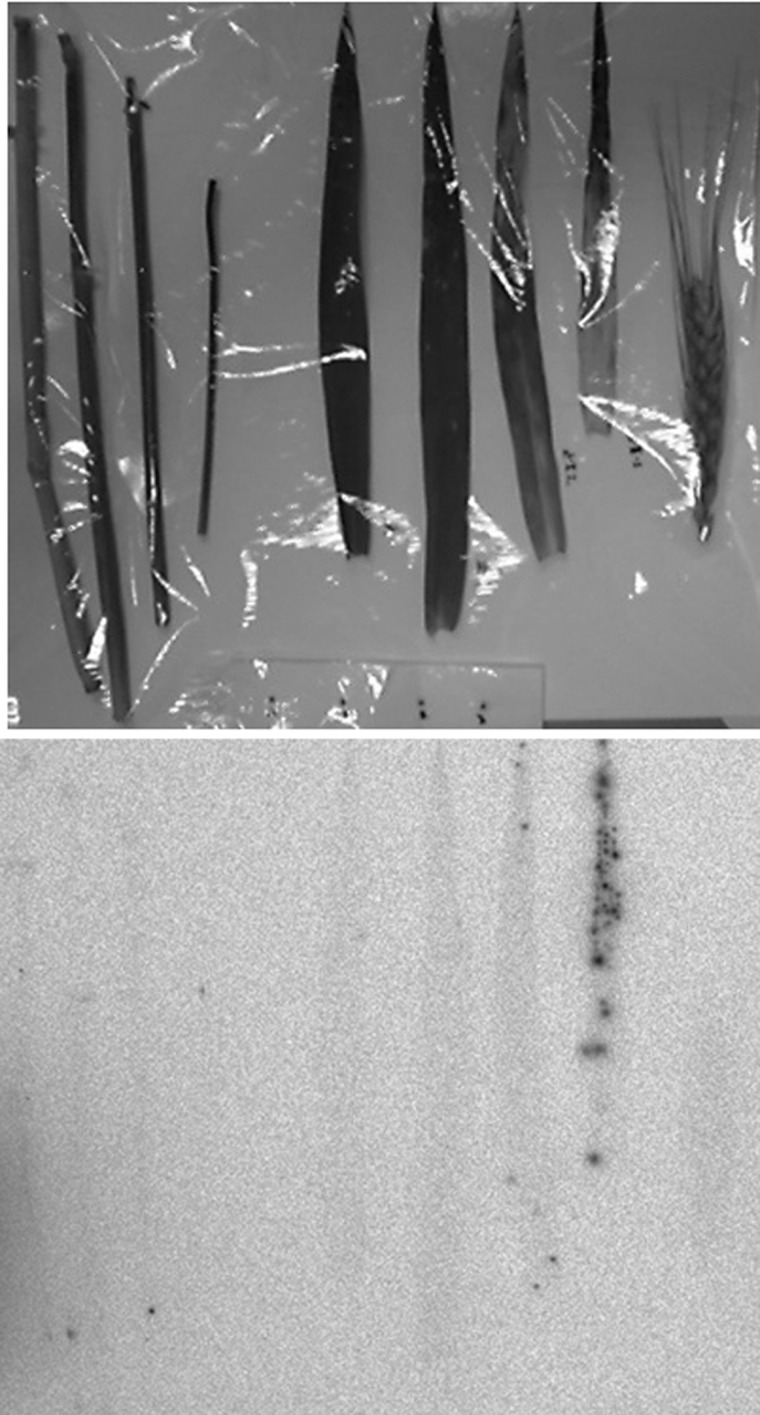
Contamination of wheat [1,2]. Two months after the accident, the wheat plant with the developed ear was harvested and a radioactivity image was taken on an imaging plate. Upper figure: photo of the wheat plant; lower figure: autoradiograph of the sample in the upper figure. Most of the radioactivity was found in an old leaf that was exposed to the air at the time of the accident; the radioactivity hardly appeared in the other tissues. This result suggested that once radioactive cesium had been adsorbed on the leaf surface, it hardly moved and was not incorporated within the leaf for transfer to the other developing tissues (K. Tanoi *et al*.).

Since the nuclear accident occurred in late winter, there were hardly any crops growing in the field, except for wheat. Two months after the accident, ears of wheat developed and the distribution of the radioactivity in the wheat tissue was determined. It was found that most of the radioactive Cs was still concentrated in the old leaves that were exposed to the air at the time of the accident (Fig. 5). The radioactivity in the leaves and ears that developed after the accident was comparatively very low, suggesting that the fallout nuclides had hardly moved from the place where they first touched, even after a few months.

When the radioactivity image of the wheat leaves was magnified, the shape of the contamination was still spot-like. When the radioactive Cs was incorporated into the leaves and had moved inside the tissue, it must have transferred along the phloem or xylem, which resulted in the parallel line images in the leaves. However, such imaging of veins was not visible in the leaves. The behavior of the radioactive Cs emitted from the nuclear accident was different from that of the so-called macroscopic Cs chemistry that we know. Because the amount of Cs deposited on the leaves was so small and carrier-free, the nuclides seem to behave like radio-colloids, or as if they were electronically adsorbed onto the tissue.

## REAL-TIME CESIUM UPTAKE IMAGING

**Fig. 6.**
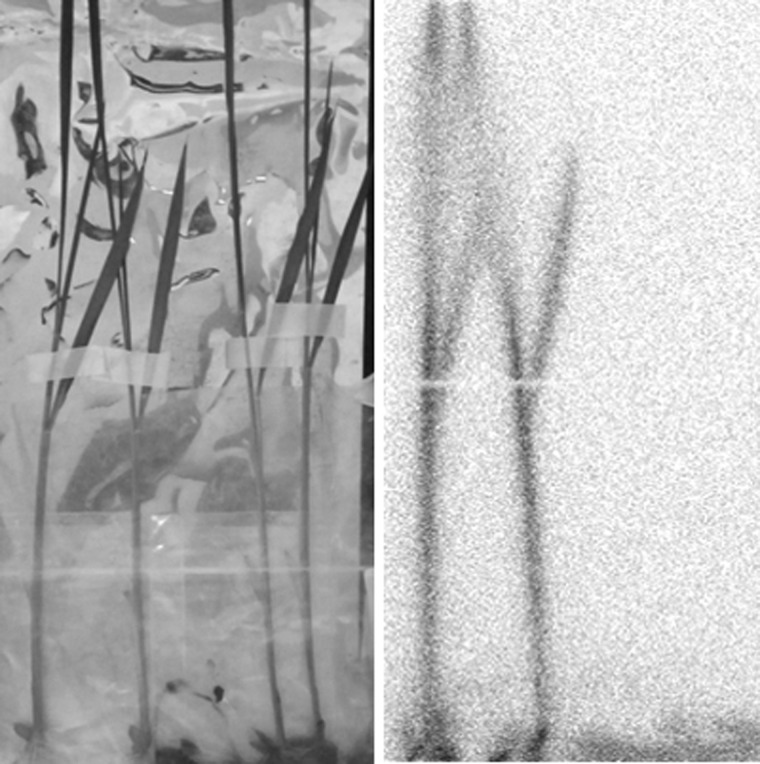
Comparison between soil culture and water culture with respect to ^137^Cs uptake [1,2]. Rice plants were grown in soil and water culture. ^137^Cs was supplied in both cultures, and the manner of uptake by the roots was compared using the real-time imaging system developed. In the case of the soil culture, the ^137^Cs was firmly adsorbed onto the soil; thus, the ^137^Cs was not absorbed by roots. However, in the case of the water culture, the ^137^Cs was rapidly absorbed by the roots. Left: photo; right: autoradiograph after 3 h. The two plants on the left of each image were grown in water culture and the two plants on the right were grown in soil (N.I. Kobayashi *et al*.).

Using the real-time radioisotope imaging system we developed, it was found that there was a discrepancy between the radioactive cesium absorption of the plant growing in the water culture and that of the plant growing in the field [1,2]. In water culture, the plants absorbed a high amount of ^137^Cs within hours, whereas in the paddy soil, ^137^Cs was trapped firmly by the soil and was unavailable for the plants (Fig. 6). In the case of water culture, ^137^Cs is dissolved as an ion; therefore, it is easy for plants to absorb. However, in the soil culture, the plant root was not able to access the ^137^Cs adsorbed to the soil. Water culture is a popular tool for studying ^137^Cs absorption by plants in the laboratory, but it should be noted that water culture provides a different result from that of soil culture, and the field study is principally based on soil culture.

## ANIMALS

**Fig. 7.**
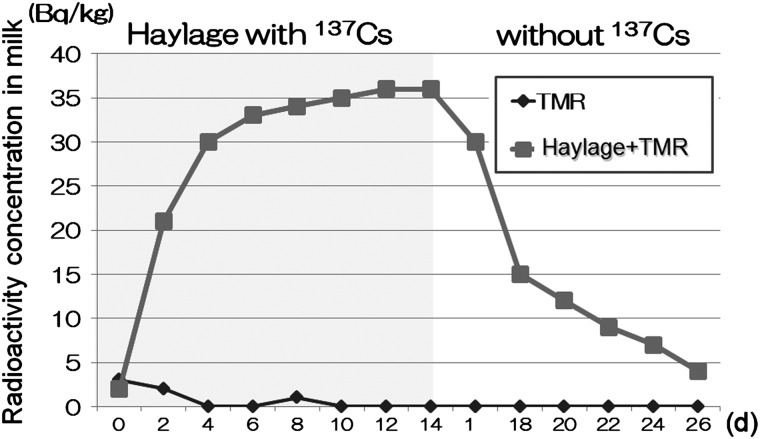
Contamination of milk [1,2]. A contaminated haylage was supplied to the dairy cattle for 14 days and the radioactivity of the milk was measured. The radioactivity of the milk was detected immediately after the supply of the contaminated haylage and reached a plateau after 14 days. Then, the supply of contaminated haylage was stopped and only non-contaminated total mixed ration (TMR) feed mixture was supplied for another 14 days The radioactivity in the milk gradually decreased and the value was close to the control value after 14 days (N. Manabe et al.).

Contaminated haylage was supplied to dairy cattle and the radioactivity of the milk was measured [1,2]. It was found that radioactive Cs was detected in the milk soon after the contaminated feed was supplied. The radioactivity of the milk reached a plateau after ∼14 days. Then, the feed was changed to a non-contaminated feed. As soon as the non-contaminated feed was supplied, the radioactivity in the milk was decreased, and the value was close to the background level after 14 days (Fig. 7). Similar results were found for animal meat, indicating that when contaminated animals are identified, it is possible to decontaminate them by feeding non-contaminated feeds. Radioactivity in the living animal was rapidly decreased after feeding it non-contaminated feeds. The biological half-life of ^137^Cs was estimated to be <100 days because of metabolism, whereas the physical half-life of ^137^Cs is 30 years.

Around the highly contaminated area in Fukushima, natural mating of pigs and wild boars is taking place, and the number of hybrid animals is increasing. Pigs and wild boars are habitually digging the surface of the soil to search for food, and they may inhale or eat a portion of the soil. In comparison, cows eat only plants. As a consequence of this difference in eating activity, radioactive Cs in pigs or wild boars is much higher than that in cows.

## DECONTAMINATION

**Fig. 8.**
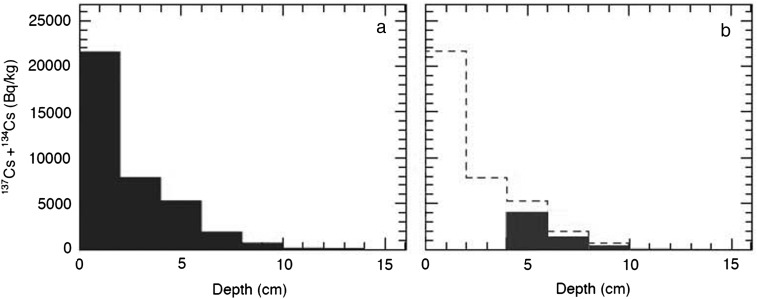
Decontamination of the rice field [1,2]. Radioactive Cs was only adsorbed onto the fine clay part of the soil; hence, it took time to sediment when water was introduced to the field and mixed with the surface soil. After mixing the surface soil with water, first the larger particles settled out, but the fine particles remained suspended in the supernatant. At this stage the fine particles suspended in the water were separated off into the ditch prepared next to the farming field, leaving most of the non-contaminated soil behind. Through this method, >80% of the radioactivity was removed: a: before treatment, b: after the removal of the supernatant. The radioactive Cs remained on the bottom or the walls of the ditch and hardly moved. When the ditch was filled in with non-contaminated soil, most of the radioactivity was shielded (M. Mizoguchi *et al*.).

To prevent radioactive Cs uptake in crops, the most effective and efficient method was found to be supplying potassium (K) as a fertilizer. Other chemicals such as Prussian blue or zeolite are very costly for agriculture. Since soil is a very important natural resource for agriculture, the removal of the surface soil cannot be compensated for by simply replacing it with other soil. It is best to eliminate only the contaminated particles in the soil. Radioactive Cs was found to be adsorbed firmly only onto the fine clay part [1,2]. When water was introduced to the field and mixed with the surface soil, it took time for the fine clay to sediment. After most of the soil component had settled out, the radioactive clay suspended in the supernatant was able to be driven into the ditch prepared at the rim of the field. Thus more than 80% of the radioactivity in the field was removed (Fig. 8).

## CONCLUSION

The results summarized above represent only a portion of our research into the Fukushima nuclear accident. The first collection of papers on this subject was published by Springer in 2013. It was made available as an open access book, free to download, so that the results of the research and studies could be widely shared among foreign and domestic researchers. We are continuing with our studies and are going to publish the second collection of papers summarizing the subsequent research results soon.

Because Japan is located in a monsoon area, with many paddy fields for growing rice plants, the climate and agricultural environment is similar to those of the other Asian countries, but is different from those in Chernobyl; therefore, it is important to gather information regarding the movement and behavior of radioactive fallout in Japan, especially as it pertains to agriculture, and to provide this information to the world.
